# The New Outlook

**Published:** 1895-08

**Authors:** 


### THE NEW OUTLOOK.

RECENTLY we read with considerable care the latest announcements of most of the American veterinary colleges, and noted with pleasure that the courses of instruction show an increase in breadth and length, and that in many cases the faculties have gained in numerical and individual strength.

Among the studies which have long been part of the curricula of the best schools, and are now more generally introduced, were observed botany, bacteriology, and meat and milk inspection. The importance of the first study in the education of an accomplished veterinarian is now generally admitted. Acquaintance with plant-life enables one to recognize immediately some common causes of injury to cattle, and the ability to classify unrecognized specimens is a most valuable part of the equipment of a country practitioner.

From bacteriological studies all the most recent advances in medicine have come. Not only is our knowledge of the cause and methods of spreading of tuberculosis, glanders, anthrax, swine-plague, hog-cholera, and many other diseases founded on work done in this field, but our still more recent knowledge of divers means of diagnosis and treatment is also the product of the bacteriological laboratory. In the domain of veterinary surgery the way is now clear for that application of bacteriological teaching which has long been made in human surgery. The meaning and true value of all this work can best be taught in the laboratories, and if out of all the schools maintaining these one student appears yearly who will devote himself, as Dr. Theobald Smith has done, to the increase of veterinary knowledge, the harvest will be abundant.

Meat and milk inspection, too, are now generally receiving the attention due them. The former is now taught practically in the pathological laboratory; but, until recently, how few veterinary graduates could, without special training, fill the position of a meat inspector?

Many of the announcements now contain the examination questions actually given the preceding year. Such a practice cannot be too highly commended. It gives a most valuable additional means of comparing the different colleges, and affords a ready index of the range and depth of the studies.

The form of the announcement deserves a word. The first impression made by taking up a well-constructed, well-worded, well-printed pamphlet cannot be other than favorable. Some colleges, unfortunately, still print ungrammatical promises of practically impossible performances, and fail to realize the damage done themselves by so doing. It seems to us, too, that advertisements take from the dignity of an announcement.

